# Clinicopathological Impact of ABCC1/MRP1 and ABCC4/MRP4 in Epithelial Ovarian Carcinoma

**DOI:** 10.1155/2013/143202

**Published:** 2013-08-19

**Authors:** Marina Bagnoli, Giovanni L. Beretta, Laura Gatti, Silvana Pilotti, Paola Alberti, Eva Tarantino, Mattia Barbareschi, Silvana Canevari, Delia Mezzanzanica, Paola Perego

**Affiliations:** ^1^Fondazione IRCSS Istituto Nazionale Tumori, Via Venezian 1, 20133 Milan, Italy; ^2^Department of Pathology, S. Chiara Hospital, Largo Medaglie Oro 9, 38122 Trento, Italy

## Abstract

Ovarian cancer is the main cause of death from gynaecological malignancies. In spite of the efficacy of platinum-paclitaxel treatment in patients with primary epithelial ovarian carcinoma, platinum-based chemotherapy is not curative and resistance remains one of the most important causes of treatment failure. Although ABC transporters have been implicated in cellular resistance to multiple drugs, the clinical relevance of these efflux pumps is still poorly understood. Thus, we examined the prognostic role of transporters of the MRP family (i.e., ABCC1/MRP1, ABCC4/MRP4) to gain insights into their clinical impacts. A case material of 127 patients with ovarian carcinoma at different stages and histotypes was used. The expression of MRP1 and MRP4 was examined by immunohistochemistry using tissue microarrays in tumor specimens collected at the time of initial surgery expression. We found an association between MRP1 expression and grading, and we observed that MRP4 displayed an unfavourable impact on disease relapse in multivariate analysis (HR = 2.05, 95% CI: 1.01–4.11; *P* = 0.045). These results suggest that in epithelial ovarian cancer, MRP1 may be a marker for aggressiveness because its expression was associated with tumor grade and support that MRP4 may play an unfavourable role in disease outcome.

## 1. Introduction

Ovarian cancer is the main cause of death from gynecological malignancies and the majority of epithelial ovarian tumors are diagnosed as advanced stage diseases [1]. Recent studies have shown that ovarian carcinoma is a complex disease that gathers molecularly different tumors sharing the localization more than biological features [2]. The management of ovarian carcinoma includes cytoreductive surgery, followed by platinum (carboplatin or cisplatin) taxane chemotherapy, which has become a standard treatment for advanced stage disease [3, 4]. In spite of the efficacy of the platinum-paclitaxel combination with responses observed in at least three quarters of the patients, the outcome is poor and responses are incomplete. The limited efficacy of chemotherapy may be dependent on the expression of defence factors which confer increased survival potential for tumor cells and, as a consequence, a multidrug resistant phenotype [5]. Therefore, an analysis of the expression by tumor cells of members of the ATP Binding Cassette (ABC) efflux transporter superfamily may be useful and could help in the choice of treatments defined on the basis of the molecular features of the tumor.

Whole-genome approaches have documented the existence of a wide family of ABC transporters, including 50 different members that can be grouped into seven distinct classes based on sequence similarities [6, 7]. Whereas it has been clearly established that ABCB1 and ABCG2 do not confer resistance to platinum compounds, selected members of the ABCC/MRP family have been implicated in resistance to platinum compounds and taxanes [8, 9]. The notion that ABC transporters are a large family of genes supports the need for novel studies directed at clarifying the relationship between less characterized transporters and resistance. Also, the recent knowledge achieved by integrating biochemical and molecular approaches indicates that the investigation of expression of ABC transporters in large collections of tumor specimens should be pursued in an attempt to elucidate the role of such factors in tumor biology. In fact, ABC transporter expression has been linked to tumor aggressiveness in different tumor types [10].

In a cellular study, cells resistant to platinum compounds were found to display increased levels of MRP1 and MRP4 [9]. In addition, the available evidence supports that the effects of transporters of the MRP subfamily on cell survival may be indirect, not necessarily implicating only efflux of the cytotoxic drugs [8]. In this regard, MRP4 has been involved in signalling pathways which activate prosurvival mechanisms by virtue of its capability to pump cyclic nucleotides outside the cells [11]. 

Based on this background, to document the role of MRP1 and MRP4 in the clinical setting in ovarian carcinoma, we investigated the role of MRPs as prognostic markers for this tumor using archival material from tumor specimens collected at surgery from epithelial ovarian carcinoma (EOC) patients. 

## 2. Material and Methods

### 2.1. Patients, Tissue Specimens, and Pathologic Data

This study was performed on a tissue microarray (TMA) containing a series of formalin-fixed, paraffin-embedded tissues collected at surgery before any chemotherapeutic treatment from 127 consecutive EOC patients who underwent surgical resection at the S. Chiara Hospital in Trento between 1992 and 1999 [12]. Histological sections and paraffin blocks were obtained from the Hospital Department of Pathology. Clinical data and follow-up information were available from the Unit of Gynecologic Oncology as specified in institutional follow-up procedures. The use of tissue blocks and patient records was approved by the Institutional Review Board. All patients gave informed consent for the therapy and for the use of specimens for research. 

Table 1 summarizes the patients' clinicopathological characteristics. The average age of the patients was 58 years. Tumor staging was in accordance with International Federation of Gynecology and Obstetrics (FIGO) criteria; 31% of patients had stage I-II and 68% of patients had stage III-IV disease. Most tumors were of serous histotype (65%). Forty-five percent of the cases were low grade (well moderately differentiated) and 52% were high-grade tumors (poorly differentiated, undifferentiated). Primary treatment for all patients was surgery, and based on the extent of residual disease after primary surgery, the patient population was divided into three groups: no evident disease (NED), minimal residual disease (mRD, residual tumor smaller than 1 cm) and gross residual disease (GRD, residual tumor equal/grater than 1 cm), [13]. Residual disease after surgical debulking was defined for 112 patients and was optimal (not evident disease or below 1 cm) in 52% of cases and equal/greater than 1 cm in 36% of cases. After surgery, 119 patients received front-line treatment with standard platinum-based therapeutic schedules (platinum without taxanes; platinum and paclitaxel) according to the time of accrual/year of diagnosis; two patients were treated with other chemotherapeutic agents, eight patients (all stage I) received no chemotherapy, and one patient had information missing. Response to therapy was available for 99 patients. Response was based on data from medical records, instrumental evaluation, and Ca 125 levels and was scored as complete (57 cases), partial (disease reduced by 50%) (21 cases), or absent (21 cases) according to the WHO standard criteria [14]. Remission was defined after completion of first-line treatment as disappearance of all clinical, radiological, and biochemical evidence of EOC. Follow-up time was based on patient date of death or the last information available in the medical records. The median of follow-up period for all patients was 89 months. Time to progression (TTP) was calculated as the time in months from the date of surgery until the first evidence (clinical, instrumental, or biological) of disease progression.

### 2.2. Immunohistochemistry

MRP expression was examined by immunohistochemistry (IHC) on formalin-fixed, paraffin-embedded sections of EOC TMA. Briefly, after xylene deparaffinization and alcohol rehydration, sections were subjected to antigen retrieval in 10 mM, pH 6.0, citrate buffer at 95°C for 6 min and 15 min in autoclave for MRP4 and MRP1, respectively. Endogenous peroxidase was quenched by incubating the slide with 3% H_2_O_2_ for 10 min. After washing, slides were incubated in saturating solution (PBS 1% BSA) for 30 min at room temperature (RT), followed by 1-hour incubation at RT with primary rat monoclonal anti-MRP1 (MRPr1, MONOSAN) or MRP4 (M4 I-80, MONOSAN) antibody at a 1 : 20 dilution. After washing, slides were incubated for 30 min at room temperature with biotinylated anti-rat secondary antibody (1 : 200, Dako S.p.A, Milan, Italy) followed by HPR-streptavidin for 30 min at room temperature. The peroxidase reaction was developed with 3, 3′-diaminobenzidine (Dako), and sections were counterstained with hematoxylin. Slides incubated with secondary antibody alone provided negative controls. The IHC experimental protocol was set up using the IGROV-1 cell line, which expresses MRP1 and MRP4 proteins as observed through Western blotting.

Staining was recorded by a semiquantitative grading system. Samples were defined as positive when 10% of cells displayed reactivity. Slides were evaluated by two independent observers blinded to patient characteristics and outcome. All cases with discrepant evaluations were discussed during observation with a double-headed microscope, and a consensus was reached.

### 2.3. Statistical Methods and Data Analysis

Subsets of patients were grouped based on similar clinical-pathological parameters (see Tables 1, 2, 3, 4, and 5). Fisher's test or *χ* ^2^ test was used to analyze the distribution of MRP positive cases in relation to clinical and pathological category variables. The effects of MRPs' expression on time to progression (TTP) were investigated first by univariate analysis through the inspection of Kaplan-Meyer curves and differences between curves were assessed for statistical significance using the log-rank test.

A Cox univariate model was used to estimate the hazard ratio (HR) for each prognostic variable considered. Multivariable analysis using a Cox regression model was used to evaluate the prognostic impact of MRPs expression in the context of concomitant effects of other known prognostic factors. Frontline therapy was used as a stratification factor accounting for its possible nonproportional effect. The *P* values of all statistical tests were 2 sided. For all analyses, differences were considered significant at *P* lower than 0.05. Analyses were performed with the GraphPad Software version 3.03 and R statistical language (URL: http://www.R-project.org/).

## 3. Results

### 3.1. Expression of MRP1 and MRP4 in Primary Epithelial Ovarian Carcinoma

**Figure 1:**
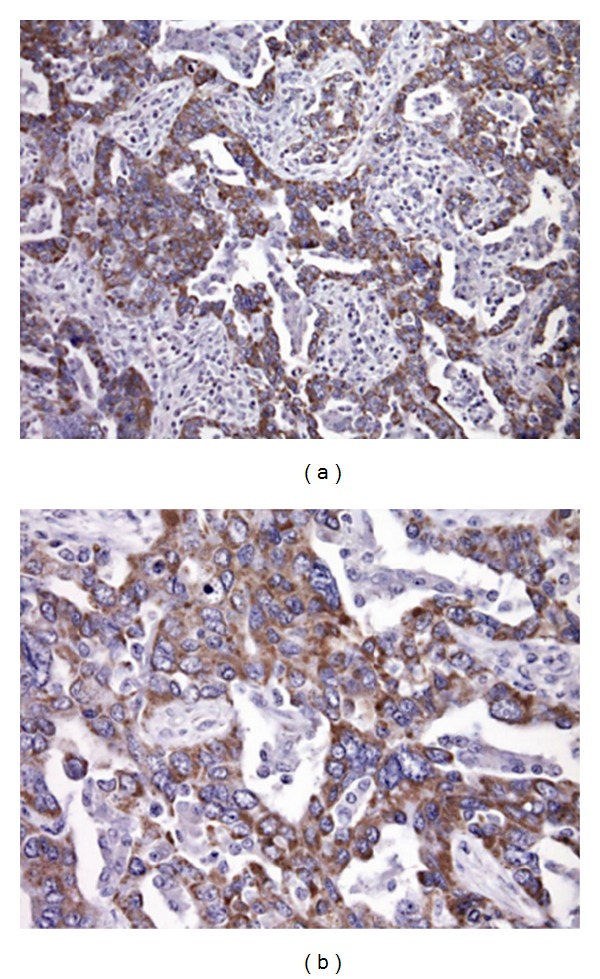
MRP1 immunostaining in specimens of ovarian carcinoma. Immunostaining decorates most of tumor cells of an ovarian carcinoma. MRP1 displayed a cytoplasmic localization consistent with cellular localization at subcellular membranes. Cytoplasmic immunoreactivity frequently displayed a granular aspect.

**Figure 2:**
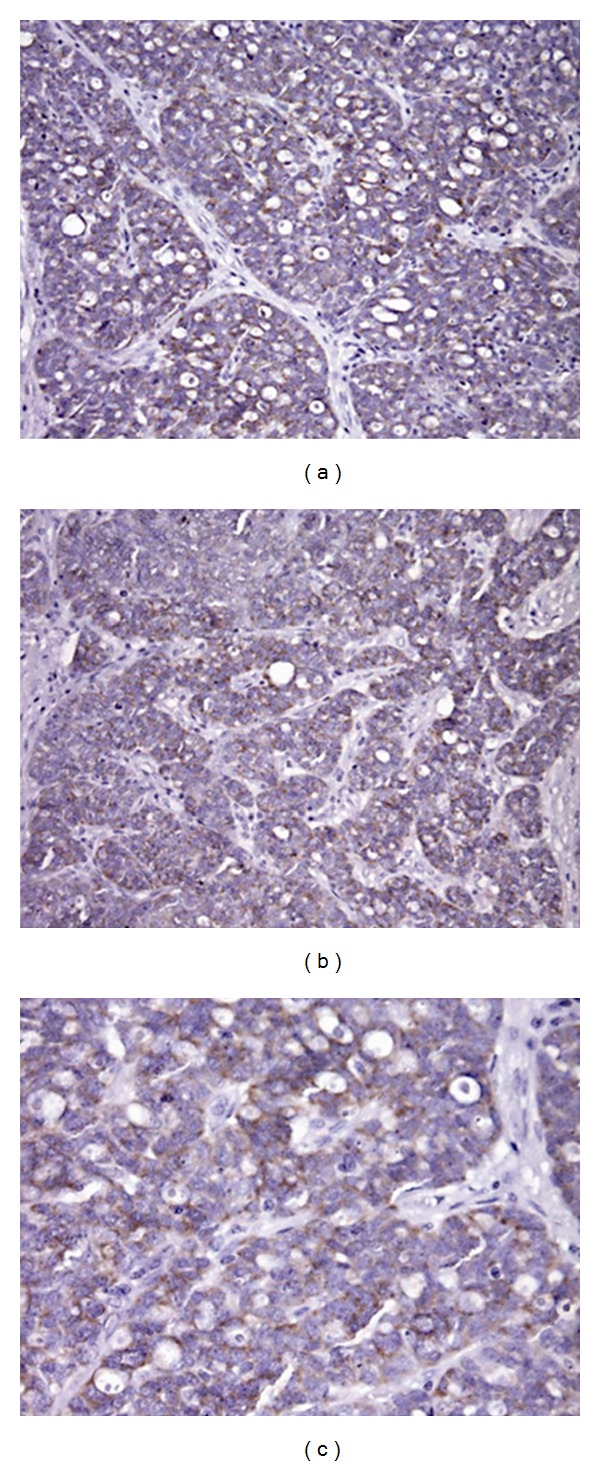
MRP4 immunostaining in specimens of ovarian carcinoma. MRP4 displayed a cytoplasmic localization consistent with cellular localization at subcellular membranes. Cytoplasmic immunoreactivity frequently displayed a granular aspect.

To investigate the expression of ABC transporters in ovarian cancer tissues, tumor specimens were analyzed by IHC. In tumor specimens, the MRP1 and MRP4 specific staining mainly displayed a cytoplasmic localization consistent with localization at subcellular membranes (Figures 1 and 2). The cytoplasmic immunoreactivity frequently displayed a granular aspect, similar to what is observed in cell lines using immunofluorescence (data not shown). Overall, in specimens, the staining intensity was more intense for MRP1 than for MRP4, a feature that may reflect tumor biology.

 Thirty percent of assessable tumors were MRP1 reactive and 23% were MRP4 reactive. An analysis of the association between MRP1 or MRP4 expression and known clinical prognostic factors indicated a statistically significant association between tumor grade and MRP1 expression (*χ* ^2^ = 8.47; *P* = 0.037, Table 2). No correlation with other clinicopathological characteristics was observed.

MRP1 and MRP4 expression was concurrently assessable for 101 cases. Among MRP1 negative tumors, only 14% (10 out of 84) were MRP4 positive, whereas among MRP1 positive cases 47% (13 out of 36) were MRP4 positive (*χ* ^2^ = 10.5; *P* = 0.0011). Then, the fraction of tumors positive for both transporters was 14% (13 out of 101 cases; Table 3).

### 3.2. Relationship between Prognostic Variables and MRP1 or MRP4

In an attempt to define the prognostic impact of MRP1 and MRP4 on TTP, we performed univariate analyses to seek for associations. Since a low number (*n* = 9) of tumors with a G1 grade was available in the present case material, analyses were carried out without including such cases (Table 4). As expected, the analyses indicated that known clinical prognostic factors (such as advanced stage III-IV versus I-II), histotype (serous versus others), and residual tumor after surgical debulking were associated with shorter TTP in high-grade EOC informative cases (Table 4). Using this approach, we found that neither MRP1 nor MRP4 was significantly associated with TTP. 

We then applied multivariable Cox regression model with MRP1 and MRP4 expression adjusting for all known clinical and pathological prognostic factors. In this model, well-established clinical prognostic factors (stage and residual disease) maintained their unfavourable prognostic impact. MRP1 did not display any prognostic impact, whereas that of MRP4 was unfavourable (HR = 2.05, 95% CI: 1.01–4.11; *P* = 0.045).

## 4. Discussion

Preclinical evidence supports that the expression of MRP1 and MRP4 is increased in ovarian carcinoma cells characterized by resistance to platinum compounds [9]. Furthermore, we have previously shown that lung cancer CD133^+^ cells, which are spared by cisplatin treatment in xenografted lung cancer models, are enriched in ABC transporters, including MRP1 and MRP4 [15]. Thus, the present study was designed to examine the expression of MRP1 and MRP4 on archival material from EOC patients with known clinical history. IHC was chosen as a method with the specific aim to set up procedures potentially useful for prospective clinical studies first, and then for routine analysis of tumor specimens. Also, we elected to use TMA because they provide a high-throughput technique for the molecular profiling of tissue specimens strengthened by the availability of sequentially sliced samples from the same master block to test the expression of the relevant macromolecules [16].

Given the function of drug transporters and their capability to extrude from cells toxins, drugs, and physiological substrates [8], a clear definition of their prognostic role is not straightforward to achieve. The available evidence supports that the ABC transporters MRP1 and MRP4 were shown to display an unfavorable prognostic impact on neuroblastoma [17, 18]. Also, in this tumor type, a prognostic value for MRP1 has been clearly documented by carrying out a prospective study [18].

In the present retrospective study, the prognostic impact was evaluated in terms of progression-free survival which, by being an earlier end point than overall survival, was considered more appropriate when modelling the prognostic significance of drug transporters which are expected to influence treatment outcome and to control drug efficacy. Indeed, overall survival may be dependent on a multiplicity of variables, not necessarily on expression of drug transporters. In fact, EOC patients resistant to first-line chemotherapy (around 30%) as well as patients that experienced disease relapse after successful first-line chemotherapy (around 70% of sensitive patients) [19] receive further chemotherapy regimen, including different agents with a variable pattern of recognition by ABC transporters.

Considering disease relapse as the clinical end point of our analysis, an unfavorable role for the expression of MRP4 was also observed in this case material, whereas this behaviour was not found for MRP1. Here, we also found an association between MRP1 expression and tumor grade. This observation is at least in part in keeping with a paper reporting expression of MRP1 in aggressive ovarian carcinoma [20]. In fact, significantly increased MRP1 protein expression was observed in high-grade tumors, similar to the present study. However, the case material used by Faggad and coauthors is not representative of the standard clinical behaviour of this disease regarding survival rate and prognostic impact of clinical variables (e.g., stage). Here, we did not find an association between disease stage and MRP1, and patients with higher expression of MRP1 protein did not exhibit significantly decreased overall survival (data not shown). Although our data validate only in part the previous findings, the discrepancies may be due to the different features of the two case materials. However, the bottom line of these findings is that less differentiated cells express the MRP1 protein. Of note, MRP1 levels have been shown to correlate with grading in untreated hepatocellular carcinoma [21]. The association between MRP1 and grading suggests an aggressive nature of the MRP1 expressing tumors. Since less differentiated tumors are expected to be endowed with the greatest proliferation potential, thereby being chemoresponsive, the unfavourable role of MRP1 may be difficult to assess. Thus, the prospective collection of a more homogeneous case material with respect to this pathologic parameter (i.e., grading) may be helpful in designing the prognostic role of MRP1 in disease relapse.

## 5. Conclusion

In the present study, using a case material containing ovarian carcinoma specimens, we found an association between MRP1 and grading and we observed that MRP4 displayed an unfavourable role in disease outcome. However, additional effort is required to better understand the precise mechanism by which MRP1 and MRP4 may influence tumor biology and drug resistance in an attempt to rationally develop therapeutic options including combination therapies based on the use of modulators of ABC transporters [22] or drugs that are not substrates for such efflux pumps [8].

## Figures and Tables

**Table 1:** Patient's clinical characteristics.

Characteristics	Patients (*n* = 127)	
	N°	%
Age, years		
(mean, median 58; range 23–84)		
Tumor histotype		
Serous	83	*65 *
Undifferentiated	12	*10 *
Clear cell	12	*10 *
Endometrioid	12	*10 *
Mucinous	6	*4 *
Others + mixed	2	*1 *
Tumor stage (FIGO)		
I	24	*19 *
II	15	*12 *
III	64	*50 *
IV	23	*18 *
Not available	1	*1 *
Tumor grade		
1: well differentiated	9	*7 *
2: moderately differentiated	48	*38 *
3: poorly differentiated	53	*42 *
Undifferentiated	12	*10 *
Not available	5	*3 *
Amount of residual disease		
NED	47	*37 *
<1 cm	19	*15 *
>1 cm	46	*36 *
Not available	15	*12 *
Frontline treatment		
None	8	*6 *
Platinum without taxanes	81	*64 *
Platinum/paclitaxel	35	*28 *
Other or not available	3	*2 *
Response to frontline treatment*		
Complete	57	*46 *
Partial	21	*18 *
No response	21	*18 *
Not available	21	* 18 *

Abbreviations: FIGO: International Federation of Gynecological and Obstetrics staging system. NED: not evident disease. *Untreated patients are not included.

**Table 2:** Patient's clinical and pathological characteristics according to MRP1 and MRP4 expression as assessed by immunohistochemistry.

Clinical parameters Total (*n* = 127)	MRP1 expression						MRP4 expression					
	Negative (*n* = 84)		Positive (*n* = 36)		Missing (*n* = 7)	P*	Negative (*n* = 80)		Positive (*n* = 24)		Missing (*n* = 23)	P*
	N°	%	N°	%			N°	%	N°	%		
Age, years						*0.245 *						0.971
≤55	40	*75 *	13	*25 *	2		33	*77 *	10	*23 *	12	
>55	44	*66 *	23	*34 *	5		47	*77 *	14	*23 *	11	
Tumor histotype						*0.617 *						0.686
Serous	53	*68 *	25	*32 *	5		52	*74 *	18	*26 *	13	
Undifferentiated	9	*82 *	2	*18 *	1		7	*87 *	1	*13 *	4	
Clear cell	8	*67 *	4	*33 *			10	*83 *	2	*17 *		
Endometrioid	8	*67 *	4	*33 *			6	*67 *	3	*33 *	3	
Mucinous	5	*100 *			1		3	*100 *			3	
Others	1	*50 *	1	*50 *			2	*100 *				
Tumor stage (FIGO)						*0.178 *						0.172
I + II	30	*79 *	8	*21 *	1		30	*83 *	6	*17 *	3	
III	40	*69 *	18	*31 *	6		36	*78 *	10	*22 *	18	
IV	13	*57 *	10	*43 *			13	*62 *	8	*38 *	2	
Missing	1						1					
Tumor grade						**0.037**						0.821
1	7	*78 *	2	*22 *			5	*83 *	1	*17 *	3	
2	37	*80 *	9	*20 *	2		32	*74 *	11	*26 *	5	
3	27	*55 *	22	*45 *	4		31	*74 *	11	*59 *	11	
Undifferentiated	9	* 82 *	2	*18 *	1		7	*87 *	1	*13 *	4	
Missing	4		1				5					
Amount of residual disease						*0.32 *						0.29
NED	33	*75 *	11	*25 *	3		31	*82 *	7	*18 *	9	
<1 cm	12	*67 *	6	*33 *	1		10	*62 *	6	*38 *	3	
>1 cm	27	*60 *	18	*40 *	1		26	*70 *	11	*30 *	9	
Missing	12		1		2		13				2	

NED: not evidence of disease. *P values: evaluated on MRP1 and MRP4 expression and available clinical and pathological parameters.

**Table 3:** MRP1 versus MRP4 expression in EOC as assessed by immunohistochemical staining.

MRP1 expression	MRP4 expression			*χ* ^2^ (*P*-value)
	Negative 80	Positive 24	Not available 23	
Negative				
84	63 (86%)*	10 (14%)	11	
Positive				
36	15 (54%)	13 (47%)	8	**10.536**
Not available				
7	2	1	4	*0.0011 *

*Raw percentages are reported and refer to the only case valuable for both MRPs.

**Table 4:** Univariate analysis of the prognostic impact of clinical covariates and MRPs on progression-free survival in high-grade EOC.

	P*	HR	(95% CI)
Stage			
III-IV versus I-II	*<0.0001 *	7.78	3.85–15.7
Histotype			
Serous versus others	*0.011 *	1.9	1.16–3.12
Surgical debulking			
SOD versus OD	*<0.0001 *	3.4	2.09–5.52
Age at diagnosis			
>55 versus ≤55	*0.48 *	1.17	0.76–1.8
MRP1 expression			
Positive versus negative	*0.86 *	0.96	0.6–1.53
MRP4 expression			
Positive versus negative	*0.27 *	1.35	0.8–2.28

*P value determined using log-rank test; HR: hazard ratio; CI: confidence interval; SOD: suboptimal debulking (residual disease >1 cm), OD: optimal debulking (residual disease not evident or <1 cm).

**Table 5:** Multivariable analysis of the prognostic impact of clinical covariates and MRPs on progression free survival in high-grade EOC.

	P*	HR	(95% CI)
Stage			
III-IV versus I-II	*<0.0001 *	10.5	3.56–30.8
Histotype			
Serous versus others	*0.96 *	0.98	0.47–2.05
Surgical debulking			
SOD versus OD	*0.016 *	2.22	1.16–4.26
Age at diagnosis			
>55 versus ≤55	*0.07 *	0.57	0.3–1.3
MRP1 expression			
Positive versus negative	*0.11 *	0.58	0.31–1.13
MRP4 expression			
Positive versus negative	*0.045 *	2.05	1.01–4.11

*P value determined using log rank test; HR: hazard ratio; CI: confidence interval; SOD: suboptimal debulking (residual disease >1 cm), OD: optimal debulking (residual disease not evident or <1 cm).
